# Feeling the force: Changes in a left-lateralized network of brain areas under simulated workday conditions are reflected in subjective mental effort investment

**DOI:** 10.1371/journal.pone.0198204

**Published:** 2018-06-18

**Authors:** Tobias Otto, Fred R. H. Zijlstra, Rainer Goebel

**Affiliations:** 1 Department of Work and Social Psychology, Faculty of Psychology and Neuroscience, Maastricht University, Maastricht, the Netherlands; 2 Department of Cognitive Neuroscience, Faculty of Psychology and Neuroscience, Maastricht University, Maastricht, the Netherlands; 3 Department of Neuroimaging and Neuromodeling, Netherlands Institute for Neuroscience, Amsterdam, the Netherlands; Universiteit Gent, BELGIUM

## Abstract

Investing mental effort is costly, and the investment has to be matched by a reward to make a person engage in task performance. However, the neural structures underlying the continued management of mental effort are not known. Previous work has identified left-lateralized structures, most prominently the left anterior Insular Cortex (aIC) as regions implied in post-hoc evaluation and also anticipation of mental effort investment. We present a study aimed at identifying neural structures that are sensitive to changes in both task load and fatigue-induced state load. Sixteen healthy participants performed an n-back task before and after a fatigue-inducing day in a helicopter simulator or a free day. Subjective mental effort ratings showed an interaction of the effects of both task and state load changes, with a reduced effect of task load during the fatigued state. Testing for the same interaction effect in a whole-brain functional MRI data, we found a left-lateralized group of clusters in aIC, the anterior cingulate cortex, the dorsal striatum and frontal eye field and M1. We discuss the possible role of these areas and also the relevance of our findings in the light of the proposed opportunity cost model of mental effort.

## 1. Introduction

### 1.1 Scope of the study

Mental effort is the psychological concept that connects motives for goal-directed behavior to the acts of self-regulation that enable the actual pursuit of the goal [1,2]. A person that has decided to pursue a goal will regulate his or her information processing and arousal in line with the demands of the task ahead. The investment of mental effort goes paired with a subjective feeling of strain [3].

Recent models of effortful mental performance consequentially treat mental effort as a cost factor [4,5]. A core assumption is that the amount of mental effort a person invests is continuously monitored. A favorable outcome of an evaluation of the amount of required mental effort in relation to the expected reward is necessary for a person to invest effort.

There has been progress in identifying the neural systems that perform both the evaluation of rewards and decide whether the effort/reward balance is favorable (see [4]). However, the neural structures underlying the continued management of mental effort investment have remained elusive. In the current study, we thus aim to identify neural structures that are influenced by factors both task- and state related, as both of these are known to influence the subjectively experienced amount of mental effort investment.

To begin, we will briefly summarize a number of theoretical considerations concerning the different factors that influence the subjectively experienced amount of mental effort. These theoretical considerations make it clear that the current body of literature is not conclusive:

Most experimental designs utilize only variations in task difficulty to change (assumed) levels of mental effort investment, but ignore the state of the participant as the other influential factor. We propose an experimental design to include both manipulations of task difficulty and participant state while monitoring mental effort investment.

### 1.2 The concept of mental effort

The amount of mental effort necessary for an individual to carry out a mental task is influenced by both the task difficulty and the individual’s current state [6]. Performing a mental task, no matter how simple, requires regulative action from an individual. Together, all the regulative actions that an individual needs to perform are referred to as the mental workload. The integrated model of mental effort [6] refers to two sources of demands: The task demands and the state demands. All regulative actions that are determined by the task itself, such as the need to control information processing or responses, form the task demands. These can be varied by changes in task complexity. In such a case, a discrepancy between the desired performance and the actual performance can signal the need to invest additional mental effort. All factors necessitating regulative action to maintain a psycho-physiological working state that allows task performance, however, form the ‘state demands’. Any mismatch of the actual and the required state of the individual, as for example due to fatigue, increases the state demands. The combination of these demands forms the total mental workload that a given task poses for an individual at a specific moment. In order to meet the mental workload, an individual needs to invest an according amount of mental effort. The subjectively experienced amount of invested mental effort forms the basis for the economic decision making process in which a person decides whether the reward for a given task justifies the required amount of effort [4]. Subjectively experienced mental effort investment can be measured such as the one-dimensional Rating Scale Mental Effort (RSME; [1].

Mental laboratory tasks such as the n-back working memory task [7] allow for increments in task load for example by varying the number of items a participant needs to keep in working memory. Likewise, inducing a state such as exhaustion or intoxication will increase the state load component of the total mental workload the participant has to meet, and hence the necessary amount of mental effort [1]. In the current study, we are interested both in mental effort as a mediator between work load and performance, as well as in the subjective experience of mental effort investment.

#### 1.2.1 Combining task load and state load in experimental paradigms

The combined effect of changes in task- and state load on the necessary amount of invested mental effort has been shown to be less straightforward, however.

Studies using sustained performance paradigms have shown that an interaction between state- and task load can take place [8]. Matthews and Desmond for example [9] showed that fatigued participants have the tendency to withdraw mental effort investment under easy task conditions compared to demanding task conditions. In their study, participants performed a sustained simulated driving task, which in one condition included an additional, fatigue inducing task. When comparing performance on a straight road (low task load) to performance on a curvy road (high task load), fatigued participants showed a decrease in performance and mental effort investment during the low task load condition, but not during the high task load condition. As discussed by Liu and Wu [8], task-induced fatigue makes a person more susceptible to the arousal-decreasing effects of task monotony. This is especially true in the absence of any immediate performance feedback, leading to a reduction in mental effort investment to the degree that actual performance decreases.

In the context of experimental research into mental effort investment, these findings emphasize the need to include a measurement of subjectively invested mental effort, as it is not trivial to simply predict changes in mental effort investment from the task conditions. Performance itself is not a good approximation of invested mental effort, as it will only decrease after the limit of acceptable mental effort investment for the participant is reached [1].

#### 1.2.2 Neural correlates of mental effort

The neural correlates of mental effort have recently become a topic of interest in the context of economic decision making. Mental effort is seen as a cost factor, as a person’s decision to invest mental effort into either a task depends on the perception of the amount of perceived effort in relation to the associated reward [4,5]. The representation of the cost of mental effort has been located in dorsolateral prefrontal cortex (dlPFC) [10], while an accumulative tracking of the cost/reward balance has been located in the intraparietal sulcus (IPS; [11] but see Botvinick and Braver [12] for a detailed review.

As the picture regarding the neural correlates of cost/reward-based decision making related to mental effort investment becomes clearer, the neural correlates of tracking the subjectively experienced amount of mental effort are still not clearly identified [5]. In their review of the literature, Westbrook and Braver identify a number of candidate regions for the tracking of subjective mental effort experience. These areas are the anterior Insular Cortex (aIC), the Anterior Cingulate Cortex (ACC), and the dlPFC.

#### 1.2.3 Neural correlates of changing workload

The occurrence of task-load dependent modulations during tasks necessitating executive control in dlPFC, aIC and ACC have previously been observed in several experimental studies also utilizing either letter-based [13] or feature-based working memory tasks [14], but also in listening span and inspection time tasks [15], but see also Owen, McMillan, Laird and Bullmore or Radua et al. [16,17] for meta-analyses.

Next to pure manipulation of task load alone, a number of studies have also used manipulations in state load in order to evoke compensatory effort. Examples of such manipulations are sleep deprivation [18], recent (but not acute) consumption of cannabis versus sustained abstinence [19] or vaccination-induced inflammation responses [20]. All of these studies have in common that they either merely assume changes in the amount of invested mental effort or derive it from indirect variables such as performance changes. Despite these limitations, their results do provide at least an indication that aIC, ACC, dlPFC and also the striatum are affected by increases in subjectively experienced mental effort as a result of increased state load.

The current literature thus provides evidence that makes it seem plausible that state load—mediated changes in mental workload have an effect similar to that mediated by task load changes on aIC activation. Furthermore, mental effort is in the mentioned studies either inferred from performance, or measured using self-constructed scales. As argued by [1], subjective mental effort investment should be measured using validated instruments constructed for that purpose. Also, fatigue in all of these studies was induced by factors other than sustained cognitive performance.

Only one study to date uses the induction of mental fatigue by sustained, albeit short (1 hour) performance [21], showing an interaction of fatigue and task load in the midbrain during task performance.

### 1.3 Rationale for the current study

#### 1.3.1 Recent developments in modelling mental effort

In the recently proposed opportunity cost model of mental effort [4], a differentiation is made between central fatigue as a result of e.g. sleep deprivation and motivational fatigue as a consequence of mental effort investment. In case of motivational fatigue, subjectively experienced increases in task-related mental effort or fatigue are seen as motivational signals, and not as indicators of spending any actual finite cognitive resource. The crucial factor in this model is what Kurzban et al.[4] call opportunity costs, i.e. the potential reward of engaging in any other mental activity instead of task performance. Specifically named in this context is the activity of daydreaming, which carries instant reward and can be performed at any given time by allocating the divisible cognitive resources away from the task. More demanding conditions of e.g. a working memory task are predicted to carry higher opportunity costs at any given moment, as they preclude parallel day dreaming to a higher degree than easy versions of the same task. When looking at the effect of sustained performance, the model predicts that increasing time-on-task with the prospect of a fixed reward would lead to an increasingly worse cost/reward balance expressed in increased motivational fatigue. Under these circumstances, Kurzban et al. [4] predict that mental effort would be withdrawn, leading to the performance decreases witnessed in laboratory studies after prolonged performance. The buildup of (motivational) fatigue is thus thought to reflect the tendency to withdraw mental effort, and not the depletion of a limited cognitive resource.

One limiting factor in the evidence reviewed by Kurzban et al. [4] is the short (~20 minutes) duration of the experimental paradigms. Healthy people routinely carry out mental work for periods of several hours in occupational settings. While fatigue might build up, performance is routinely kept stable in accordance with task goals. A recent study using longer mental performance paradigms yields evidence that shows a separation of fatigue buildup on the one hand and motivation/performance on the other:

Gergelyfi, Jacob, Olivier and Zénon [22] used a 2-hour Sudoku task in order to build up mental fatigue. The Sudoku task was interleaved with short assessments of motivation and fatigue. The results showed that while fatigue buildup was related to a decrease in performance, neither was related to changes in motivation. Furthermore, a variation in the reward that the participants could earn for successful performance failed to show any alleviating effect on the deteriorating performance. Gergelyfi et al. [22] interpret these results as a sign that in the case of longer sustained performance, the buildup of mental fatigue and subjective loss of motivation are co-occurring, but not causally related. The authors take this as evidence for the existence of an effect of sustained mental performance on cognitive resources. A recent neuroimaging study into the effects of sustained mental performance on resting state networks provides evidence supporting this view. Esposito, Otto, Zijlstra and Goebel [23] showed that mental fatigue evoked by sustained, exhaustive performance over the course of 4 hours affected parts of the control network, amongst other brain regions. This evidence points out transient changes in the functional architecture of the healthy human brain, and it is plausible that these changes are related to changes in cognitive processing capabilities observed after sustained effortful mental performance.

Most recently, Blain, Hollard and Pessiglione [24] have shown that sustained mental performance intended to mirror the exhaustion of a full working day selectively reduces activity in the left middle frontal gyrus (MFG) during a self-regulation task involving choice impulsivity. Decrease in left MFG activation corresponded to decreased self-regulation capacity.

In summary, this evidence suggests that the prolonged investment of mental effort is not only accompanied by the subjective feeling of investing mental effort, but that it can cause transient neurophysiological effects that potentially affect subsequent performance.

#### 1.3.2 The current study

In the current study, we thus aimed to clarify which neural structures are reactive to both acute changes in task load and to the changes in state load due to mental fatigue onset after sustained performance.

In order to manipulate state demands, we incorporated two different day conditions in our study. The first condition required participants to engage in sustained mental performance: Over the course of several hours we simulated the strain a participant would normally experience on a working day.

Sustained task performance has been successfully used to induce a state of increased mental fatigue, which influences the experienced amount of required mental effort to perform mental tasks; [1,25]. In order to be able to separate this from the circadian effects, participants also were measured on a free day, on which all effortful activities were prohibited.

#### 1.3.3 Hypotheses

Hypothesis 1: We expect the effortful day treatment to result in an increased level of fatigue (measured by items from the Profiles of Mood Scale (POMS; [26]) and decreased levels of well-being (measured with four items from the revised English version of the Eigenzustands (“own state”) scale (EZ-Scale) [27]) on the afternoon of the working day, compared with the afternoon of the free day. The effects of the manipulations in task load and state load are expected to interact as shown in previous literature: in the non-fatigued state, changes in task load within the performance range of participants can be expected to lead to a linear increase in the amount of invested mental effort [1,9]. While the induction of fatigue as such will increase the state load and thus the amount of mental effort participants need to invest, this effect has been postulated to not be even across the different levels of task load, with easy task conditions being affected stronger during higher state load conditions [8]. Matthews and Desmond showed that participants would withdraw their mental effort investment selectively during easy task conditions after a fatigue-inducing treatment. However, in their experiment, participants had to rely on their own perception of their performance. The accuracy of this perception could have been affected during the fatigued state, prompting the withdrawal of mental effort. In order to rule this factor out, our paradigm is designed to provide immediate performance feedback to participants.

Hypothesis 2: We expect to find a three-way interaction between effects of the day time, the day treatment and the task condition on subjectively experienced mental effort: Higher task load will lead to increased experience of mental effort investment. However, the size of the task load effect will be reduced after the effortful day condition, as the need to invest mental effort will increase disproportionally in the easy task condition.

Variations in the task load on working memory tasks affect brain activity in a number of cortical and subcortical regions. Reviews of studies using either the n-back task in particular [16] or a variety of mental tasks [17] showed an effect of increasing task load in the bilateral insula, in several bilateral foci in the frontal and parietal cortices and in the bilateral striatum. As we are employing a standard variant of the n-back task, we expect to find an increase of brain activation due to increasing task load in locations similar to the ones reported in these two reviews.

Hypothesis 3: Increased task demands during n-back-task performance will be reflected in increased brain activation in areas previously identified to react to increases in mental load, in particular the aIC, striatum, frontal and parietal areas.

The main research interest of this study is to combine changes in task- and state load in the same participants in order to identify brain areas that show sensitivity to the combined effect of both manipulations. A brain region underlying the experience of mental effort investment should hence show the same three-way interaction as the subjective ratings of mental effort. The literature gives us reason to believe that aIC, ACC, dlPFC and the striatum play a role in the experience of mental effort.

An earlier study into the neural correlates of mental effort evaluation also suggests that it might be in particular the left aIC that plays an important role in the post-performance evaluation of invested effort [28]. In this earlier study, participants performed short blocks of an n-back working memory task. The task had three levels of difficulty (1-, 2- and 3-back). After each block, the participants had to rate their experienced mental effort investment on the Rating Scale Mental Effort (RSME; [1]) and the task difficulty on a similar visual analogue scale (VAS). This contrast of subjective mental effort rating versus task difficulty rating was chosen to compare similar task-related evaluations using similar instruments, with only the subjective, self-referenced effort investment differing between the two scales. Only a cluster of voxels in left aIC showed increased activation during mental effort evaluation compared to during difficulty evaluation.

Hypothesis 4: Activity in aIC, ACC, dlPFC and striatum will be influenced by changes in task and state load in a similar way as the subjective ratings of invested mental effort, namely with a reduced effect of task load in the fatigued condition.

## 2. Methods

### 2.1 Participants

20 healthy adult (18+) participants were recruited from the student body. [Mean age 23.3 years; 8 male]. Participants were invited only after being screened for any condition that would exclude participation in MRI research. Participants provided written informed consent prior to the experiment. MRI–naïve participants were invited to participate in the piloting scans in the planning phase of the experiment. This was done in order to minimize the effect of the unfamiliar and potentially stressful MRI environment on participants, as we assumed that this would interfere with the effects of our experimental manipulation.

All participants were introduced to the working memory task and the rating interface before the start of the experiment. Participants were compensated with vouchers conform to faculty rules. The compensation did not include any performance-dependent component. A total of 4 participants had to be excluded from the analysis due to excessive motion in at least one of the imaging runs (see *analysis* for detailed criteria), leaving the data 16 valid participants for the analysis (mean age 24, 1 years; 5 male).

### 2.2 Procedure

The experimental procedure was approved by the institutional review board (Ethical Review Committee Psychology and Neuroscience, Maastricht University) under registration number ECP_70_04_05_2008_4.

Participants arrived at the facilities around 09:30h, (+- 1h). Previously, they were instructed to get their normal amount of sleep, and not to exceed moderate caffeine levels in the morning (maximum of two cups of coffee for habitual users not less than one hour pre-experiment).

For the first MRI session, the participants were then placed in the MRI scanner. Scanner-naïve participants were accustomed to the scanner 1–2 weeks prior to the experiment in order to reduce novelty effects or stress due to being exposed to the scanner for the first time.

**Fig 1:**
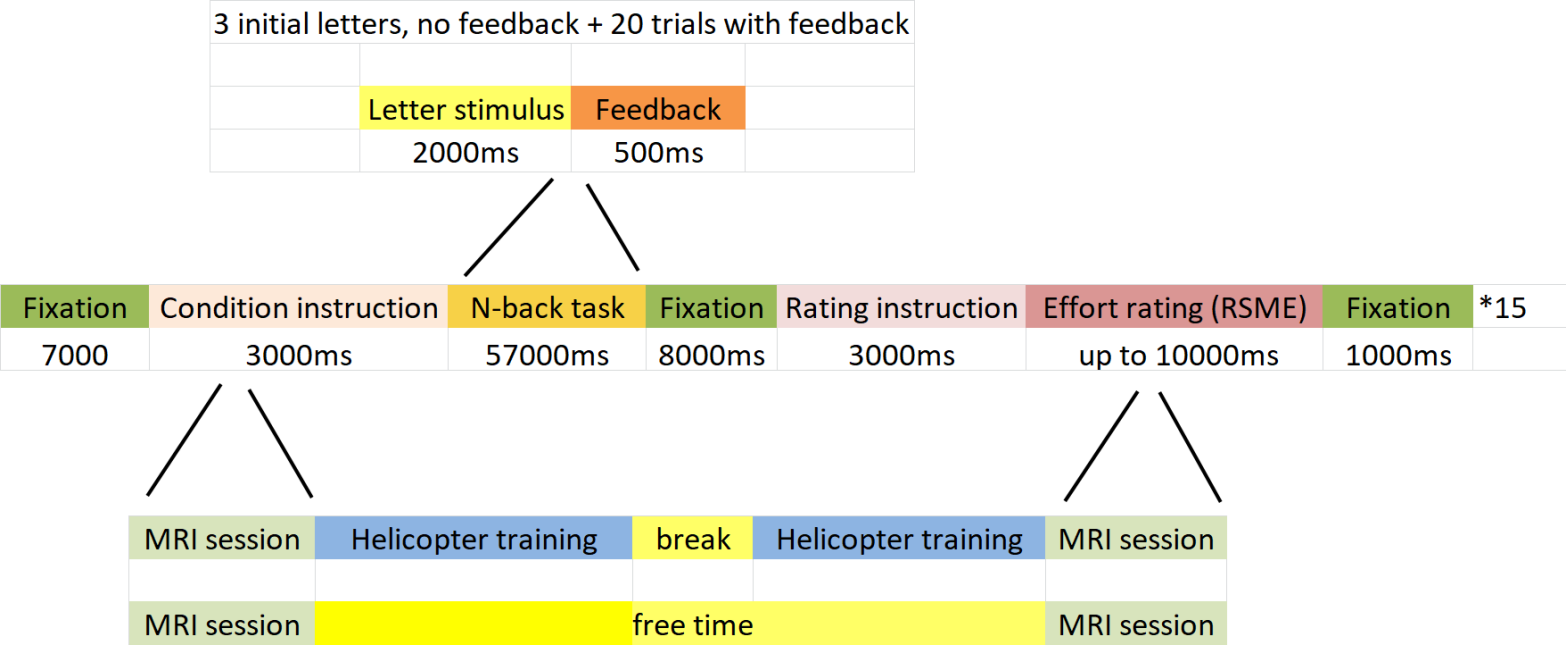
Schematic depiction of the experimental design. Top: a single n-back trial middle: a block of 20 trials, separated by fixation from the related RSME rating; bottom: the simulated working day and the free day with the MRI sessions and either helicopter training or free time.

While whole brain functional scans were acquired, participants performed a version of the n-back task [7]. Participants had to memorize letters appearing on a screen and indicate through a button press during the 2000ms letter presentation time if those letters were identical to the letter 1, 2, or 3 trials back, depending on the block condition. Each block of the n-back task consisted of 20 trials. A block would start by an on-screen information stating the block condition (1-, 2- or 3-back) for 3000ms. After the information screen, 3 consecutive letters were presented for 2000ms as initial target letters, without requiring a response. This was followed by the first of the 20 trials. Each trial consisted of the presentation of a letter (stimulus duration 2000ms) during which participants had to also give the response. After the end of the presentation/response time, participants would be presented for 500ms with either a green “CORRECT” for a correct button press or with a red “ERROR” for a wrong or absent button press. This was done in order to enable participants to adjust their effort expenditure directly after making a mistake. The next trial would start immediately after the end of the feedback. After the last feedback screen, a fixation cross would be presented for 8000ms. This was followed by a text instruction screen for the RSME effort rating for 3000ms. Subsequently, the rating scale would be presented for 10000ms, during which the participants would have to move the cursor on the scale and indicate their response by a button press. There was a 8000ms fixation period before the start of the next block instruction (see Fig 1 for a visual representation of the experimental design). Each of the three n-back conditions was presented five times, in a quasi-randomized order. Performance was measured as the number of correct button presses within the 2000ms letter presentation/response window. The task and the RSME were programmed in E-Prime (Psychology Software Tools, Inc., US). They were presented using E-Studio on a Windows XP PC connected to a MRI compatible optic system consisting of a projector and mirror goggles. Task and rating input was collected via an MRI compatible optical 2-button Joystick (Current Designs Inc., Philadelphia, USA). Participants trained the handling of the Joystick for a brief period before the experiment by marking values on a VAS analogue to the one used in the actual experiment.

After this first MRI session, participants either underwent a 4h training session in the university’s helicopter cockpit mock-up or spend the same amount of time with self-chosen, low-effort activities. The training treatment was designed to induce the exhaustion level of a demanding work day. The helicopter training session consisted of a short theoretical instruction on helicopter take-off procedures and a practical part of trying to perform a takeoff procedure according to the presented guidelines. At around 16:00h, participants returned to the MRI lab. The participants performed the same n-back paradigm as in the morning session. All participants both underwent the free day treatment and the work day treatment in quasi-randomized order. Time between the days varied between 2 and 8 days, based on the scheduling possibilities for the fMRI lab.

### 2.3 Measurements

Fatigue was assessed with four items from the POMS [26] as used for example by Sonnentag, Binnewies and Mojza [29]. Well-being was evaluated with four items from the revised English version of the Eigenzustands (“own state”) scale (EZ-Scale) [27], as used by Sonnentag and Zijlstra [30]. All items were rated on five-point-scales. Fatigue and well-being have been demonstrated to be an estimate of experienced strain [31].

Mental effort expenditure was rated using the RSME [1]. Participants were able to use an optical joystick device to move the cursor along the scale in the fMRI scanner. Performance on the task was measured in terms of errors made and reaction time (RT) to detect any unintended decreases in performance.

Magnetic resonance imaging was performed on a Siemens Allegra 3T head scanner (Siemens AG, Erlangen, Germany).

Anatomical imaging was carried out with a standard ADNI T1 weighted sequence, Voxel size1 cubic mm; flip angle = 9 deg; TR = 2250ms; TE = 2.6ms. Whole brain Echo-Planar Imaging (EPI) was performed using the following parameters: Matrix size 64x64; slice thickness 3,5mm; Slice order descending and interleaved; no gap; FOV 224x224mm; TE = 30ms; TR = 2000ms. 840 volumes were collected per run. Slice orientation was tilted 30 degrees backwards in order to minimize susceptibility artifacts in the orbitofrontal regions [32]

### 2.4 Analysis of behavioral data

Behavioral data was analyzed using SPSS 19. A mixed model analysis of the RSME scores was carried out in order to reflect the nested structure of the data. Factors included time-of-day (morning; T1, versus evening; T2); the day activity (working day, abbreviated WD, versus free day, abbreviated FD) the n-back condition (1–3) and the session order of the four session. The session order was included in the model to correct for possible learning effects that can occur in the n-back task across testing sessions even after hundreds of trials [33]. The factors were centered and a model with interaction terms was build.

Performance in terms of the number of errors and RT was analyzed in a mixed model using the same factors as for the analysis of the RSME data.

Well-being and fatigue items from each participant were pooled separately to form sum scores for the four respective time points (morning and afternoon of the working day and the free day). A2x2 ANOVA using day activity and time-of-day as within-subject factors was calculated. The effects per level of day treatment and time-of-day were then compared using a paired-samples t-test, comparing the morning and afternoon scores per each day and also the two afternoon scores. One participant reported a misinterpretation of the directionality on some of the items which lead to a number of missing values on the scales. Well-being and fatigue data from this participant was excluded from the behavioral analysis. Fatigue data of the morning of the free day was not saved for one participant; hence fatigue data for this participant could only be contrasted for the other time points.

### 2.5 Imaging data treatment

Analysis of fMRI data was performed in BrainVoyager QX 2.3 (Brain Innovation BV, Maastricht, The Netherlands). Anatomical images were individually preprocessed by inhomogeneity correction and extracranial noise filtering. The data was subsequently transformed into stereotactic space [34]. The transformed anatomical scans from all participants were then averaged into a single anatomical data set used as background for the visualization of group analyses.

The first three volumes of the functional scans were discarded because of magnetic saturation effects. The functional scans were preprocessed by slice scan time correction, motion correction and high pass filtering. High pass filtering was performed using a General Linear Model (GLM) approach with a Fourier basis set which was adjusted to regress the time course for predictors with up to 2 sine/cosine cycles per run and eventual linear trends out of the data. Volume Time Course (VTC) files were calculated for each of the 4 runs. Data of four participants showed translation/rotation exceeding 3mm/deg in at least one of the four runs. Those datasets were excluded from further analysis. Data from the remaining 16 participants was entered into the further analysis as described below.

### 2.6 Analysis of the imaging data

The E-Primer script for BrainVoyager (Hester Breman, Brain Innovation B.V.; 2009) was used to extract the timing information of the single conditions from the E-Prime protocol files for each separate run. This timing information was used to build a design matrix. The single boxcar predictor time courses were adjusted for the shape and delay of the hemodynamic response by convoluting them with a two-gamma-function [35]. Predictors for the translation/rotation of the participant’s head were derived during the motion correction of the functional data and added in the design matrix.

A random effects (RFX) GLM was computed for the runs of all remaining 16 participants. Task predictors spanned from the first letter of the n-back task to the end of the response window of the last letter of that n-back task. To identify brain areas that react to changes in task load, we computed a contrast over all four measurement points between 1-back and 3-back task execution. The resulting activation map was adjusted to a single-voxel threshold of *t* (15) = 5.24 (*p* < 0.0001). As the RSME–scores revealed an interaction of task load and state load (see results); we proceeded to identify brain areas in which such a specific three-way-interaction would also take place. Therefore, we computed an interaction contrast in which the difference between the difference of 1-back and 3-back in the morning and the difference of 1-back and 3- back in the afternoon of the working day was tested. We then subtracted the same difference calculated over the free day from this contrast in order to remain with voxels which would show the interaction effect stronger on the working day. This was done to correct for circadian effects, which we assumed to be equal on both days. The resulting activation map was adjusted to a single-voxel threshold of *t* (15) = 2.95 (*p* < 0.01).

Both maps were subsequently corrected for multiple comparisons by using the Cluster Threshold estimation plugin of BrainVoyager. This plugin runs a Monte-Carlo-Simulation extension [36] in order to determine the minimal cluster size given a user-defined confidence level, which was set to alpha = .05. The resulting minimal cluster size for the simple task load contrast map was 8 voxels, while it was 12 voxels for the three-way-interaction map. Locations of the surviving supra-threshold clusters of active voxels were identified using a microatlas of the human brain [37].

## 3. Results

### 3.1 Self reports

One participant reported a misinterpretation of the scales for exhaustion and well-being. Data from this participant was excluded from this analysis. There was a clear effect of the day activity on self-reports of both fatigue and well-being. The ANOVA for fatigue showed a significant interaction of day condition and time-of day (*F* (1, 14) = 19.12, *p* < .001). Subsequent paired-sample t-tests showed that fatigue was rated higher in the afternoon of the working day than in the morning (*M* / *SD*: 9.47 / 3.11 and 6.27/ 3.13; *t* (14) = -4.13, *p* < .001). It was also rated higher in the afternoon of the working day when compared to the afternoon of the free day (*M* / *SD*: 5.40 / 2.92; *t* (14) = 5, *p* < .0001), thereby confirming hypothesis 1. Fatigue was actually rated higher in the morning of the free day when compared to the afternoon of the free day (*M* / *SD*: 6.07/ 3.08 and 5.07 / 2.73; *t* (13) = 2.39, *p* < .03). The ANOVA for well-being showed a significant interaction of day condition and time-of day (*F* (1, 14) = 15.02, *p* < .002). Subsequent paired-sample t-tests showed that well-being was rated higher in the morning of the working day than in the afternoon (*M* / *SD*: 16.53 / 2.59 and 14 / 3.72; *t* (14) = 4.11, *p* < .001). There was no difference in well-being on the free day. Well-being was rated higher in the afternoon of the free day (*M* / *SD*: 16.8 / 1.82) than in the afternoon of the working day (*t* (14) = -3.53, *p* < .003). These results show that the state manipulation worked as predicted in hypothesis 1. Furthermore, fatigue was actually reduced over the course of the free day, which might indicate recovery from work stress unrelated to the experiment.

### 3.2 Mental effort scores

**Fig 2:**
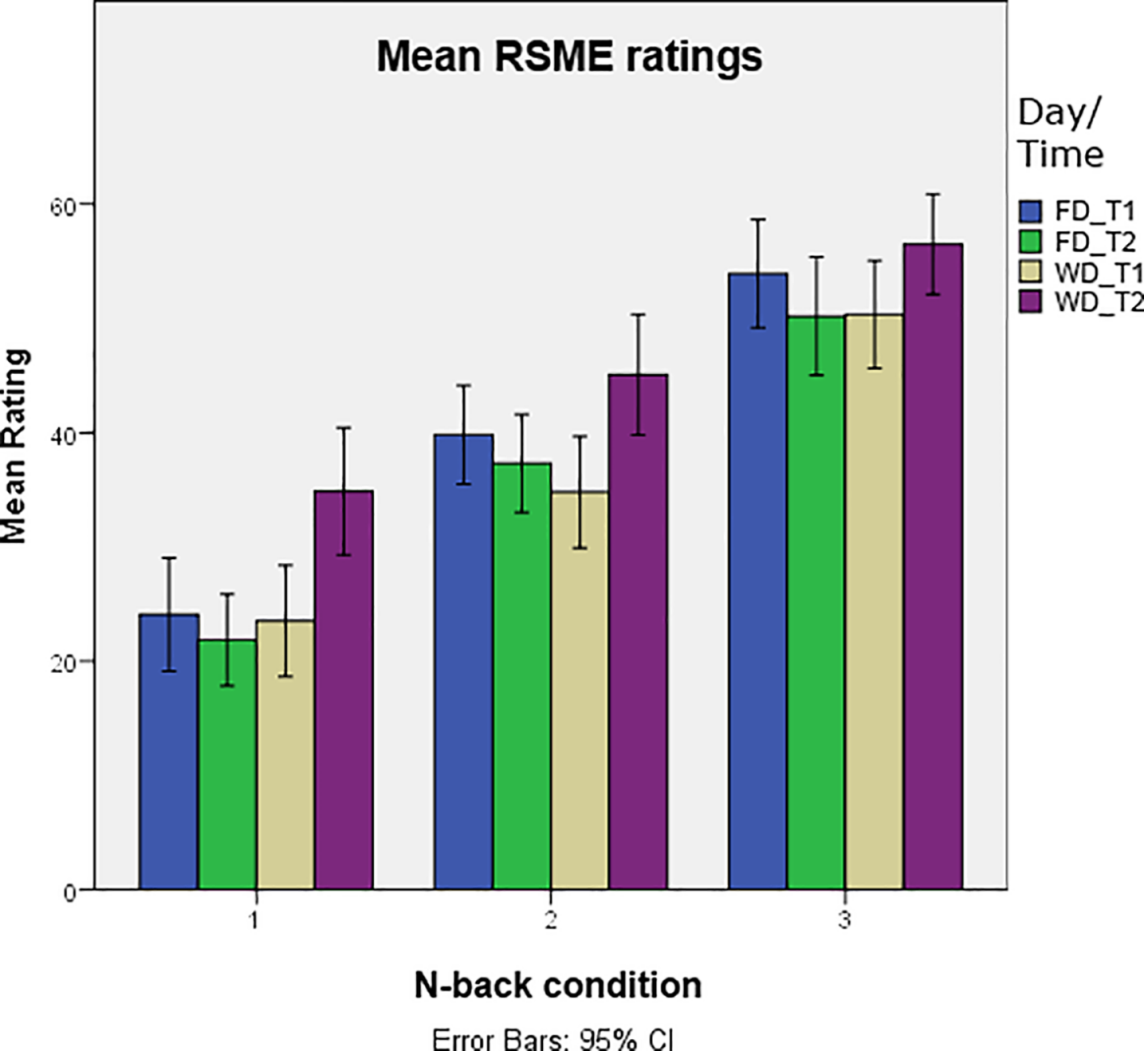
Group means of the RSME scores over the four time points and the three n-back conditions. The effect of the three different n-back levels showed an interaction with the day activity and the time of the day: An increased task load on the n-back task lead to increased self-ratings of mental effort investment. This effect is reduced at the evening of the work day, as task difficulty did not influence the experienced amount of mental effort as strongly as at the other time points. In particular, the most simple n-back condition required more effort. Error bars represent 95% confidence intervals.

We found a significant three-way-interaction between the n-back condition, the day treatment (FD/WD) and the time of the day (T1/T2), *F* (1,695) = 7.91, *p* < .005), see also Fig 2. Simple main effects per condition in the evening, contrasting the factor day treatment, revealed an effect of day treatment on the RSME ratings (1-back: (*F* (1, 159) = 20.34; *p* < .0001; 2-back: (*F* (1, 98) = 6.40; *p* < .013 and 3-back *F* (1, 68) = 4.21; *p* < .04). This is in line with our expectations: The reported amount of mental effort investment is influenced by the n-back condition and by our fatigue manipulation. Furthermore, the effect of task load changes after the induction of fatigue by prolonged performance (on the evening of the working day but not in the mornings nor in the evening of the free day), thus confirming hypothesis 2.

### 3.3 Task performance

All participants were able to perform the task successfully (group mean 1.27 errors per 20 trials, group SD 1.58). An effect of task load on the number of errors (*F* (1, 449) = 119, 87; *p* < .0001), RTs (*F* (1, 433) = 278, 35; *p* < .0001) and RSME ratings (*F* (1, 371) = 265, 96; *p* < .0001) was observed. There was no significant simple main effect of the day condition in the evening on error rates (*F* (1, 57) = .11; *p* < .737) or RTs (*F* (1, 68) = .46; *p* < .501). There was a significant simple main effect of the day condition in the evening on RSME ratings (*F* (1, 154) = 4, 69; *p* < .032). The RT data did, however, show a similar three-way interaction as the RSME data, in which the effect of the n-back task condition was reduced after fatigue induction (*F* (1, 849) = 9,64: *p* < .002. There was no significant three-way-interaction of task load, time-of-day and day treatment on errors (*F* (1, 915) = 1.69; *p* < .194). Simple main effects per condition in the evening, contrasting the factor day treatment, revealed no significant differences neither in errors nor in RTs for neither 1-back (*F* (1, 95) = .91; *p* < .342 and *F* (1, 60) = .335; *p* < .565) nor for 2-back (*F* (1, 24) = .446; *p* < 511 and *F* (1, 18) = .21; *p* < .732) nor 3-back (*F* (1, 36) = 1.83 *p* < .185 and *F* (1, 64) = .49; *p* < .487). See supplementary materials S6 and S7 Figs for an overview of the error and RT results.

### 3.4 Imaging results

The fMRI data revealed a robust effect of changes in task load over a wide range of brain areas. Contrasting activity during 3-back performance with activity during 1-back performance revealed a load-related increase in brain activation in several areas. Clusters that became more active during increased task load included the bilateral aIC, the bilateral MFG, several bilateral parietal areas and bilateral striatal/thalamic areas (see Table 1 for details). These areas have been reported earlier in a meta-analysis of brain activation during n-back performance [16]. An exception is the angular gyrus; however, a more recent meta-analysis of studies of general cognitive effort [17] does report this area as well. Our task thus evoked an increase in brain activation in line with earlier reports of the effect of cognitive effort, confirming the third hypothesis.

**Table 1:** Clusters showing the main effect of n-back condition. Coordinates represent peak coordinates.

	Peak Tal. coordinates					
Location	x	y	z	*t*	*p*	vol
AnG R	30	-67	37	10.09	0.00	5742
PRG/ inferior frontal gyrus, opercular part	51	8	37	8.77	0.00	3560
aIC/Operculum R	30	23	4	10.50	0.00	2135
MFG R	27	-1	52	9.02	0.00	3160
Caudate nucleus medial/ventral anterior Thalamic nucleus R	12	8	7	7.37	0.00	1405
SFG M B	-6	8	49	12.19	0.00	4296
medial dorsal thalamic nucleus R	9	-19	13	7.41	0.00	964
ventral anterior thalamic nucleus L	-9	-10	10	6.57	0.00	423
Caudate nucleus medial/ventral anterior Thalamic nucleus L	-15	2	7	7.23	0.00	943
MFG/SFGL L	-30	-7	52	7.96	0.00	1523
Supramarginal Gyrus L	-42	-46	40	13.29	0.00	5960
aIC/Operculum L	-33	20	1	8.47	0.00	1219
Middle Frontal Gyrus (MFG) L	-48	20	34	12.48	0.00	2039
PRG L	-45	2	31	7.72	0.00	585

Contrast showing 3-back > 1-back condition of the n-back task. Volume counted in number of anatomical voxels. Abbreviations: ANG = Angular gyrus; PrG = Precentral gyrus

As a second step, we computed a contrast analogue to the 3-way interaction between task and state load we found in the mental effort scores. This contrast represents the difference between the 1-back and the 3-back condition of the afternoon of the working day minus the difference between 1- and 3-back in the morning of the working day. Furthermore, we subtracted from this contrast the same contrast calculated over the free day to correct for changes that would occur due to circadian effects. Thus, the map shows clusters of voxels in which there was a significant interaction between n-back-condition and time-of-day on the working day, corrected for the occurrence of such an effect on the free day.

**Fig 3:**
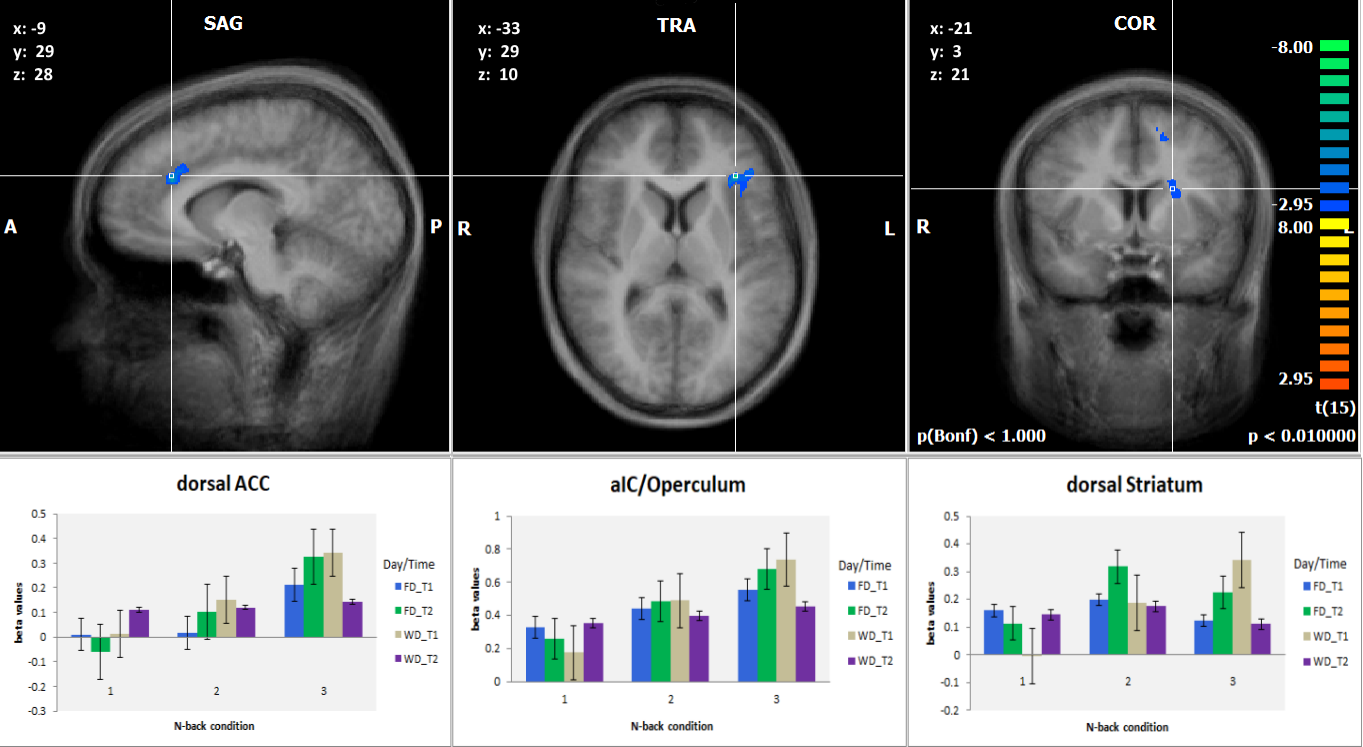
Map of 3-way clusters and associated beta diagrams. Top: Clusters in dorsal ACC, anterior Insula and dorsal striatum that showed the same 3-way interaction as the behavioral measurements of mental effort. Bottom: Beta weights of the different clusters during task performance. Note the reduction of the influence of changing n-back task load at the evening of the working day, WD T2. Betas represent average values per clusters. Beta plots are for illustrative purposes only. Error bars represent standard errors.

Clusters showing the described 3-way interaction were found in three locations hypothesized in H4: One cluster was situated in left aIC, extending into the left operculum. A second cluster was found in the left dorsal ACC (also referred to as the anterior midcingulate cortex based on more recent receptor-based neurobiological model [38]). A third cluster was found in the left dorsal striatum (caudate nucleus, stretching across the internal capsule to the putamen; see also Fig 3 for details of ACC /aIC/ /striatum). These results largely confirmed our fourth hypothesis. We did not, however, find any surviving clusters in the dlPFC. Three further clusters where found in areas not covered by H4: In the precentral gyrus/primary motor cortex (M1), the posterior medial superior frontal gyrus (SFG) and in the left lateral SFG (see Table 2 for peak coordinates of all surviving three-way clusters); for clusters depicted from all directions as well as for figures and beta plots of the posterior medial SFG and left lateral SFG clusters see supplementary materials S1–S5 Figs). The peak voxels of the medial SFG cluster were found to be situated in what has been identified as the frontal eye field (FEF) using a probabilistic mapping approach with a standard localizer task [39].

**Table 2:** Clusters showing the three-way-interaction. Coordinates represent peak coordinates.

	Peak Tal. Coordinates					
Location	x	y	z	*t*	*p*	vol
dorsal ACC	-9	29	28	-5.57	0.00	551
SFGL/FEF	-18	-4	58	-4.58	0.00	955
dorsal striatum	-24	5	19	-4.65	0.00	330
AIC/Operculum	-33	29	10	-7.55	0.00	835
precentral gyrus/M1	-36	-13	55	-5.30	0.00	371

## 4. Discussion

We conducted this study in order to identify brain areas which track controlled changes in experienced mental effort investment. We tested whether the proposed reduction of the effect of task load on subjective mental effort during fatigue [8] can also be detected in the brain. We will first discuss the specific behavioral findings and single involved brain areas, before going into the more general findings and the implications of our results.

### 4.1 Effects of the experimental manipulations on behavioral variables

We were able to use changes in both task load and state load to manipulate the experienced mental effort expenditure of the participants. The different difficulty levels of the n-back task caused distinct levels of mental effort investment, as shown by the RSME ratings. Furthermore, spending several hours in an exhaustive learning task did induce a state of fatigue in the participants, as documented by increased fatigue and reduced well-being in the afternoon measurement. This state change cannot be attributed to circadian effects, as the comparison measurement on the free day afternoon did not show any reduction in well-being, and even revealed a slight decrease in fatigue compared with the morning measurements.

Under the condition of induced fatigue, changes in task load did have a different effect on subjective mental effort than during the non-fatigued state. The direction of the effect of changing task load remained the same, with a higher working memory load leading to increases in self-reported mental effort investment. However, the effect of task load was reduced in fatigued participants (See Fig 2). While the amount of mental effort investment in the 3-back condition is only slightly higher under the influence of fatigue, effort ratings for the 1-back condition increased disproportionally. This outcome confirms our Hypothesis 2 and is in line with earlier results [9]. As reasoned already in the review by [8], fatigued participants are more susceptible to task monotony under easy task conditions, and tend to withdraw their mental effort in the absence of direct feedback. In our paradigm, however, participants were provided with continuous feedback on their performance. Hence, in order to keep performance at the required level, participants adjusted their investment of effort. The effect of task monotony is further reflected in particular in the RTs. The three-way interaction of n-back condition, day treatment and time-of-day shows a blunting of the effect of task load under fatigue. The error scores seem to follow the same pattern, keeping in mind however the non-significant result of the three-way interaction analysis. We interpret these observations as that in particular the challenging nature of the three-back task made it easier for our participants to engage in the task.

### 4.2 Effects of the experimental manipulations on brain activation

Differences in activation during performance as a result of increasing task load during all non-fatigued conditions were similar to the results known from the literature [16,17]. This confirms our third hypothesis.

The main goal of the current study, however, was to identify brain areas which react to the interaction of task load and state load. When testing for the same specific interaction that was present in the behavioral data, clusters in aIC, ACC, lateral caudate nucleus, medial SFG, lateral SFG and the precentral gyrus were detected. All significant clusters were detected exclusively on the left side of the brain. We focus here on the regions that were indicated by prior studies (aIC/ACC/striatum), as we did not have any hypotheses regarding the involvement of the superior frontal or precentral clusters.

The involvements of aIC, ACC and the dorsal striatum are in line with earlier research that associated them with the management of mental effort in relation to task performance. In particular the involvement of aIC is in line with earlier findings regarding the selective role of the left aIC/operculum in subjective rating of mental effort investment just after task performance [28]. Also, the role of left aIC in expectancy of immanent mental effort investment has been shown previously [40]. We extend the previous interpretation by Otto et al. [28] regarding the role of left aIC in post-performance mental effort rating and propose a similar functionality of this structure during the acute experience of mental effort investment.

The aIC has, together with the ACC, been proposed to form part of a salience network [41]. In this proposed network, the aIC would receive input from frontal areas regarding the presence of attention demanding stimuli, e.g. task demands and integrate this information with the information it receives about cognitive, emotional and bodily states. Increased need for attention is mediated via the ACC to the more executive cortical areas and the dorsal striatum.

Further supporting this interpretation, several studies and meta-analyses provide evidence of aIC involvement in functions relevant to the management of mental effort. The bilateral aIC, specifically the more anterior part, has been identified as part of a network underlying domain-general maintenance of task rules and strategies [42]. Thus, aIC is involved in the representation of the actual task set, which defines the mental workload posed on the organism. Additionally, there is evidence that suggests that aIC also monitors ongoing task performance by integrating task- and performance-related bottom-up information [43].

This information is particularly relevant in the case of errors signaling the need to invest additional effortful cognitive control: Ham et al. [44] showed that increased connectivity between ACC and left aIC during errors correlated with subsequent behavioral adaptation. The ACC is known for its role in error detection, but in the authors’ interpretation, the left aIC is important for implementing the behavioral adaptation, demonstrating the need for these areas to cooperate in order to achieve behavioral adjustments to improve performance. This functional lateralization of aIC has recently been highlighted as well in a study by Späti et al. [45]. While right aIC was shown to be part of a network processing outcome salience in a gain/loss paradigm, left aIC was shown to be involved in behavioral adaptation by means of influencing cognitive control. Recent results such as in Nelson et al. [43] or Ham et al. [44], however, make it likely that left aIC also has a role in monitoring task goals and cooperates with ACC in situations where behavioral adaptation is needed.

The role of the ACC in this context has been widely discussed: Shenhav, Botvinick and Cohen [46] proposed an integrative role of, in particular, the dorsal part of the ACC in relation to the expected value of control. Westbrook and Braver [5] propose that the ACC also has a role in evaluating the reward value of an action in relation to its costs. In particular the dorsal ACC has been shown to have overlapping functions in both representing reward and costs. This region has been shown to harbor overlapping areas responsive to pain, negative affect and cognitive control in a review of 192 studies [47]. According to the authors, the evidence shows that dorsal ACC provides a link between reinforcing feedback and behavioral output. This interpretation was further supported by Vassena et al. [40] who recently demonstrated this dual functionality of the dorsal ACC. While larger areas in the ACC were shown to be active in the expectation of reward, specifically an area in left dorsal ACC also showed overlapping activation during the expectation of effort. Holroyd and Yeung [48] finally not only proposed that ACC is related to motivated response selection, but that it has a close relation to structures in the dorsal striatum implied in carrying out the chosen behavior.

The dorsal striatum has previously been identified as a modulator of activity in higher cortical regions during effortful mental performance [49]. MacDonald et al. [50] specified this modulatory role of the dorsal striatum to not facilitate general mental effort, but rather cognitive flexibility, i.e. updating target stimuli that change in terms of stimulus-response relation or relevance. Flexible updating of working memory content is an integral characteristic of the n-back task used in our experiment, which gives a plausible explanation for the involvement of dorsal striatum during task performance. The striatum in general, thus extending beyond the dorsal part reported here, has also been implied both in cognitive fatigue and effort-reward-calculations [51]. A possible underlying role of dopamine levels in the striatum, but also in the prefrontal cortex has been proposed [52].

To summarize, the brain areas that we reported have all been implied in various roles in mental task performance. Foremost, the aIC has been implied in the expectancy [40] and post-performance evaluation [28] of mental effort investment. The aIC has also been implied in a performance-monitoring and performance managing role during performance [43–45]. The dorsal ACC has been shown to fulfil a dual role in both effort and reward expectancy [40] and its additional undisputed role in performance monitoring has led Shackman et al. [47] to label dorsal ACC a critical link between performance feedback and cognitive control. The dorsal striatum has been proposed to facilitate cognitive flexibility in higher cortical areas [50]. The FEF and M1 have been proposed to be involved in stimulus salience and response facilitation [53], and have been shown to correspond to attentional load [54].

A group of adjacent frontal clusters spanned from the precentral sulcus/ primary motor cortex (M1) via the lateral SFG to the medial SFG. We did not have any hypotheses regarding these areas. Our speculative interpretation is that their changing activation levels could be related to top-down regulation of attentional and sensory motor systems as proposed by Sarter, Gehring and Kozak [53].

The left-sided lateralization of the interaction effect found in our results raises an interesting point with respect to the literature. In a recent study, Engström et al. [15] argued for a central role of the right aIC (together with bilateral ACC) in cognitive effort.

A possible explanation for the left lateralization in our results is the reliance of the process of mental effort experience on certain somatic inputs. The experience of mental effort relies on integration of several sources of information about the self, and information about bodily states is thought to be crucial for this process [55]. In this context, Gray et al. [56] have proposed the left aIC as a target site for heartbeat- evoked potentials (HEP). In their combined EEG/ECG study, Gray et al. contrasted a high-workload arithmetic task with a baseline counting task in patients with heart problems. The high-workload task proved to be more stressful than the control task. Individual stress-related changes in myocardial output were significantly correlated with changes in HEP amplitude in electrode sites close to left aIC. The authors propose that left, but not right aIC is the principal target site of afferent signals related to changes in myocardial function caused by changes in acute stress. According to Craig [57], one of the defining factors in the laterality of aIC activation is origin of the processed signal. In the case of stressful stimuli, the afferent HEP signal in left aIC would be that factor.

Our results show the changing reactivity of a number of brain areas to changes in task load due to the induction of fatigue by sustained performance over four hours. How do these relate to the main goal of this paper, the identification of brain areas monitoring mental effort investment, and how do these results relate to the predictions of the ‘opportunity cost model of mental effort’?

The functions of the involved areas as demonstrated in the literature allows us to propose that left aIC, together with left ACC and left dorsal striatum have a role in monitoring mental effort investment in relation to task goals and performance. Together, these regions perform several important sub-functions to manage mental effort investment. In this cooperation of regions, the ACC can be assumed to have a more reward-related role. According to Westbrook and Braver [5] it is the ACC that integrates effort costs and benefits to determine if a task is worth performing. During acute performance, errors signal that effort needs to be adjusted. This, logically, entails that also the previously made effort-reward calculation needs to be updated. We thus interpret the increased left aIC-ACC connectivity shown by Ham et al. [44] as a sign that ACC does under these circumstances depend on input from left aIC.

We do not claim that left aIC or any of the other identified regions represent mental effort as such or that or that their activation is linearly related to the amount of subjective experience invested mental effort. Rather, we interpret the joint modulation of the aIC, the ACC and the dorsal striatum as fulfilling a role in several sub-functions related to task management: The sense of effort investment, the occurrence of errors and the need to change effort investment either due to updated effort/reward calculations or due to a goal/performance discrepancy. The effect of task monotony during the fatigued state seemed to play an important role in shaping the pattern of our interaction. During the most basic 1-back condition, our participants had to invest more mental effort in order to counter task monotony. This increase in effort investment successfully prevented a significant drop in performance both in terms of accuracy and speed, as revealed by the simple main effect contrasts. The activation of the reported brain regions corresponds to this pattern in the sense that there are more signals that indicate an increased need for effort, which is then employed to counter the increased effects of task monotony. Likewise, having to perform the task at a more challenging level meant that, despite the need for participants to invest a higher amount of effort in total, the absence of the need to fight monotony meant that there was actually less overall demand for the “managing” functions of aIC, ACC and dorsal striatum.

Tentatively, we suggest that there are transient changes in brain functioning that underlie the increased effect of task monotony during the fatigued state. It has been shown before that sustained performance, such as on our helicopter learning task affects the coherence of the cognitive control network [23]. This finding has been interpreted by Dobryakova et al. [52] as a sign that changing levels of also striatal dopamine might play a role in cognitive fatigue, as the regions indicated in Esposito et al. [23] receive dopaminergic projections. This indicates a in the cognitive resources that are available to counter task monotony under acute fatigue induced by sustained performance.

This reduced effect of task load furthermore relates our results to the ongoing discussion about the nature of control during prolonged task performance [58]. Recently, it has been proposed that mental effort investment could be modelled in a resource-free fashion in the ‘opportunity cost model of mental effort’ [4]. In this model, the subjective feeling of effort is rather based on momentary economical and motivational decision processes than a finite resource.

Our behavioral results are not in accordance with the predictions of this model: Assuming there is no change in the availability of cognitive resources, the linear effect of changes in task load on subjective mental effort should have remained the same. Hence, the task-load-dependent possibility to spend a part of one’s cognitive resources on other mental activities such as daydreaming during task performance should still determine the opportunity costs and hence the amount of mental effort. The reduced effect of task load on both behavioral measures of mental effort and on brain activation contradicts this assumption. An alternative explanation would be that the cost signal of effort itself is amplified under conditions of fatigue and high task monotony. This explanation would however necessitate explaining, in the constraints of the opportunity cost model, why task monotony becomes amplified during fatigue caused by prolonged effortful performance.

In relation to this question, Shenhav et al. [58] have recently proposed based on the evidence by Esposito et al. [23] and the recent work of Blain et al. [24] that there might be substantial differences in the resource dependency of effort investment based on the duration of the employed manipulation. The interpretation of the results of the current study, then, is in accordance with this notion: In addition to the observed changes in brain activity induced by long-time sustained activity, also subjective self-ratings of mental effort investment are affected in a way that cannot be explained exclusively with motivational differences.

### 4.3 Limitations and future directions

While we did succeed in showing the reduction of the effect of changes in task load on behavioral measures of mental effort and reactivity of brain areas alike, an estimation of this effect would have profited from a third measurement point in the middle of the day. However, constraints in budget and system time precluded this.

Additionally, the precise mechanism of how the working day treatment would lead to the changes in fatigue, well-being and n-back task effort scores is not known. While our design was conceived in order to rule out any confounding by circadian differences, there are a number of possible confounds. We cannot rule out that the influence of the day treatment on the effort scores was mediated by well-being or other, unobserved factors. Such factors could theoretically be mediating the changes in both the behavioral data and the functional imaging data, in the sense that it is not increased fatigue, but e.g. decreased well-being that underlies the observed effects. Yet, all of these conceivable factors do have in common that they constitute an additional mental load that the participant commonly needs to meet with their mental effort investment, even after an exhausting working day.

Our manipulation was conceived to mimic the demands of an exhausting working day. Reduced well-being or increased boredom is commonly associated with the normal experience after a day at work. Also, while we did not find an effect of the interaction of time, day treatment and n-back condition on task accuracy, at effect was present for RTs.

The fact that our manipulation also influenced RTs could possibly constitute a confound for the functional data. It has been shown earlier by Yarkoni et al., 2009, that differences in RT can be liable to cause differences in BOLD amplitude [59]. However, looking at the RTs, we see that the effect of n-back condition on RT in the fatigued condition is indeed blunted, most prominently by a reduction in 3-back RT. This is accompanied by also a higher effort rating for this condition. In relation to the predictions of Yarkoni et al. (2009), this would lead to an increase in the amplitude of the BOLD response observed during the 3-back condition in the fatigued condition. Looking at our Fig 3, however, it is clear that rather the opposite is the case—the signal in our data is decreased under these circumstances.

A question that remains for future research is how long the effect of the fatiguing day treatment would persist. Is recovery a question of a mere few hours, or a night’s sleep, or does one even recover more during the course of a free day, as the reported reduction in fatigue from morning to afternoon on the free day suggest? Ideally, we would have had more measurement points during both days, and also followed the evening measurement up with at least one pre-bed time and one morning-after measurement. Again, practical limitations precluded this in the current study. However, future studies should try to determine the time course of the reported interaction between task load and state load, and the time course of the recovery to baseline. Also, while our manipulation utilized fatigue induced by sustained performance as state load manipulation, it would be interesting to see other manipulations of state load, for example in the opposite direction by means of stimulants.

In a larger scope, we echo Shenhav et al.’s [58] suggestion of a closer connection between task duration and resource dependency in sustained effortful performance. Research into this domain seems warranted from a theoretical viewpoint, as it would clarify the fundamental question of resource-dependency in mental effort investment. Furthermore, understanding the effects of effort investment on the timescale of actual working days will help model the processes present in the average working population. The relevance of such future achievements for the field of work psychology cannot be understated.

## Supporting information

S1 Fig FEF and precentral gyrus clusters and beta values resulting from the three-way-interaction. (DOCX)

S2 Fig FEF and precentral gyrus clusters and beta values resulting from the three-way-interaction. (DOCX)

S3 Fig dACC cluster in all three viewing directions. (DOCX)

S4 Fig aIC cluster in all three viewing directions. (DOCX)

S5 Fig Dorsal striatum cluster in all three viewing directions. (DOCX)

S6 Fig Overview of mean errors and RTs (in ms). (DOCX)

S7 Fig Overview of mean errors and RTs (in ms). (DOCX)
