# Supplementary figures and images for: Feeling the force: Changes in a left-lateralized network of brain areas under simulated workday conditions are reflected in subjective mental effort investment
Source: PLoS One. 2018 Jun 18;13(6):e0198204. doi: 10.1371/journal.pone.0198204 (PMC6005543; doi:10.1371/journal.pone.0198204)

**S1 Fig: FEF cluster and beta values resulting from the three-way-interaction**

**
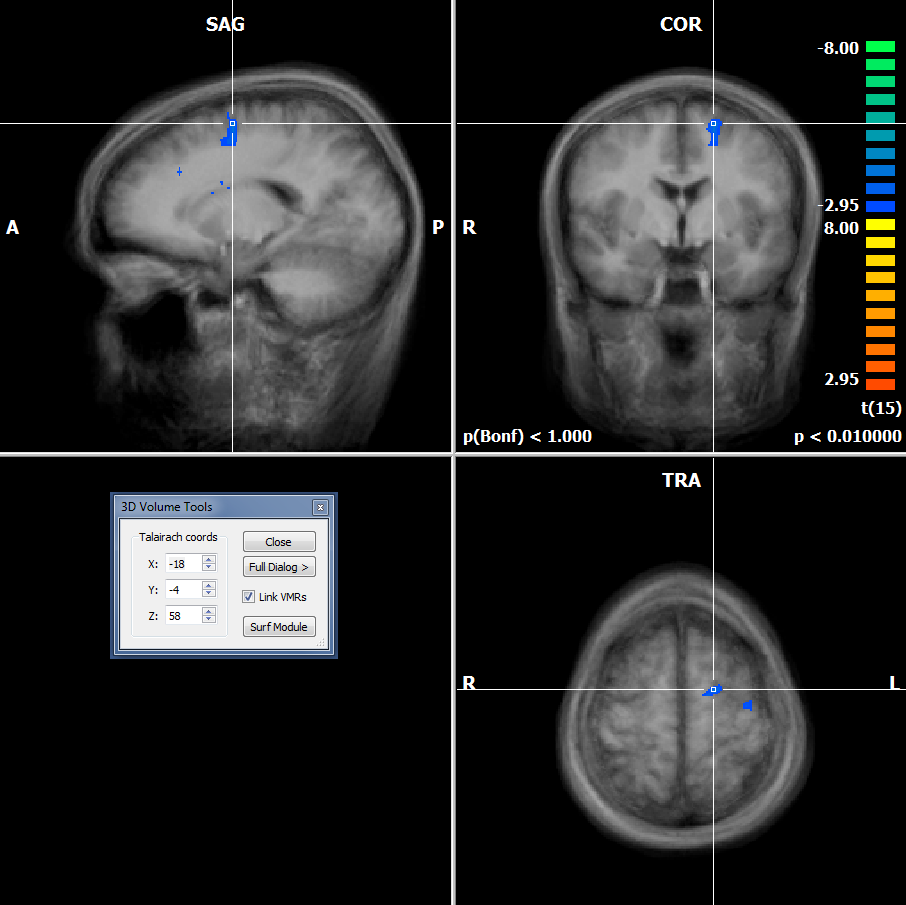
**

**
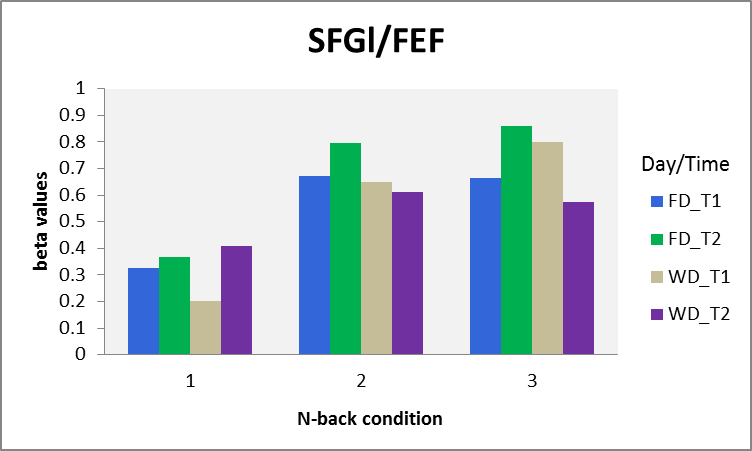
**

Supplement: S1 Fig — (DOCX) [file pone.0198204.s001.docx]

**
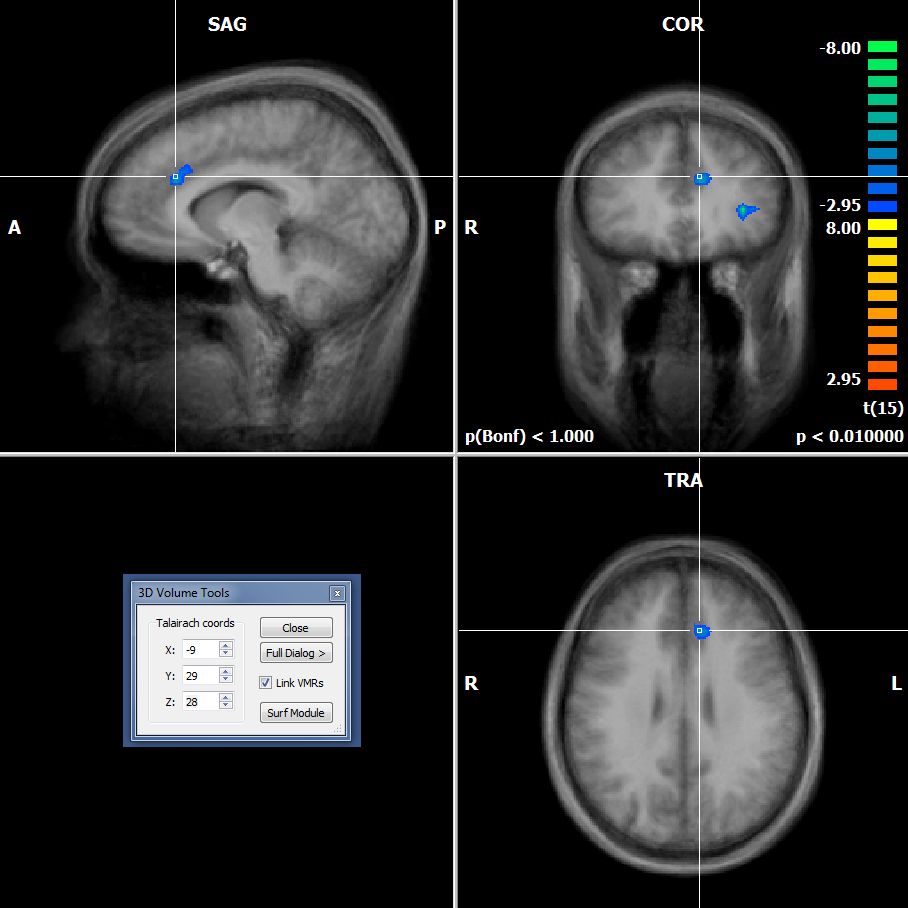
**

S3 Fig

Supplement: S3 Fig — (DOCX) [file pone.0198204.s003.docx]

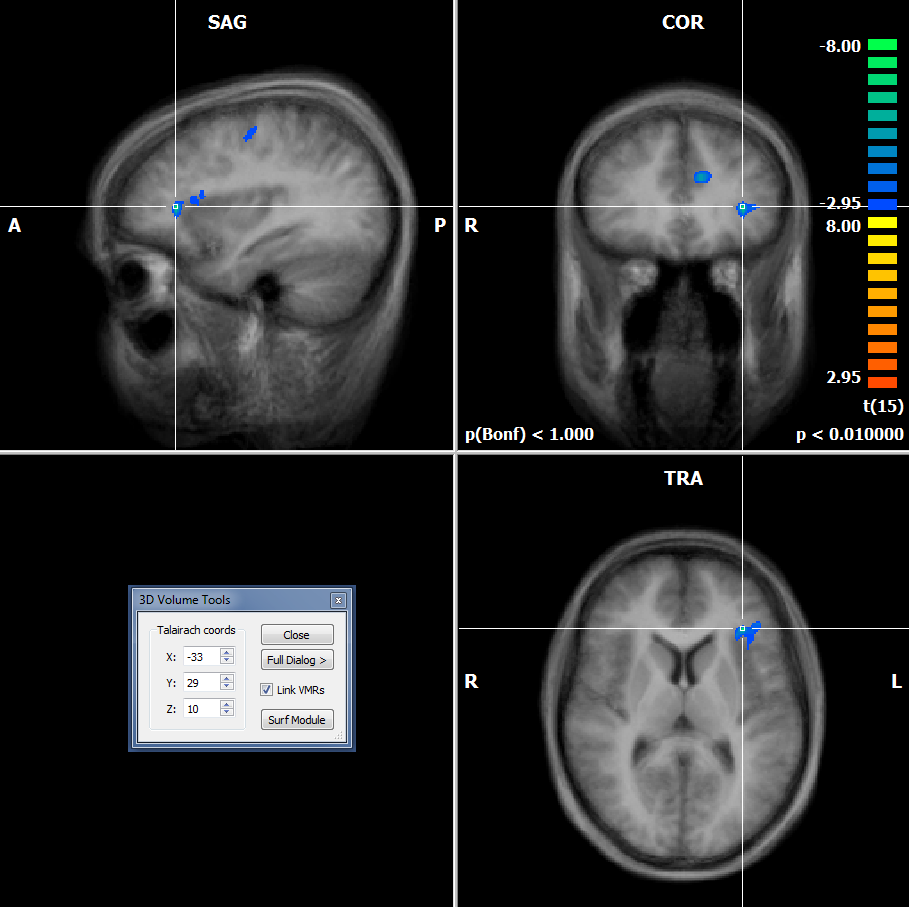
S4 Fig

Supplement: S4 Fig — (DOCX) [file pone.0198204.s004.docx]

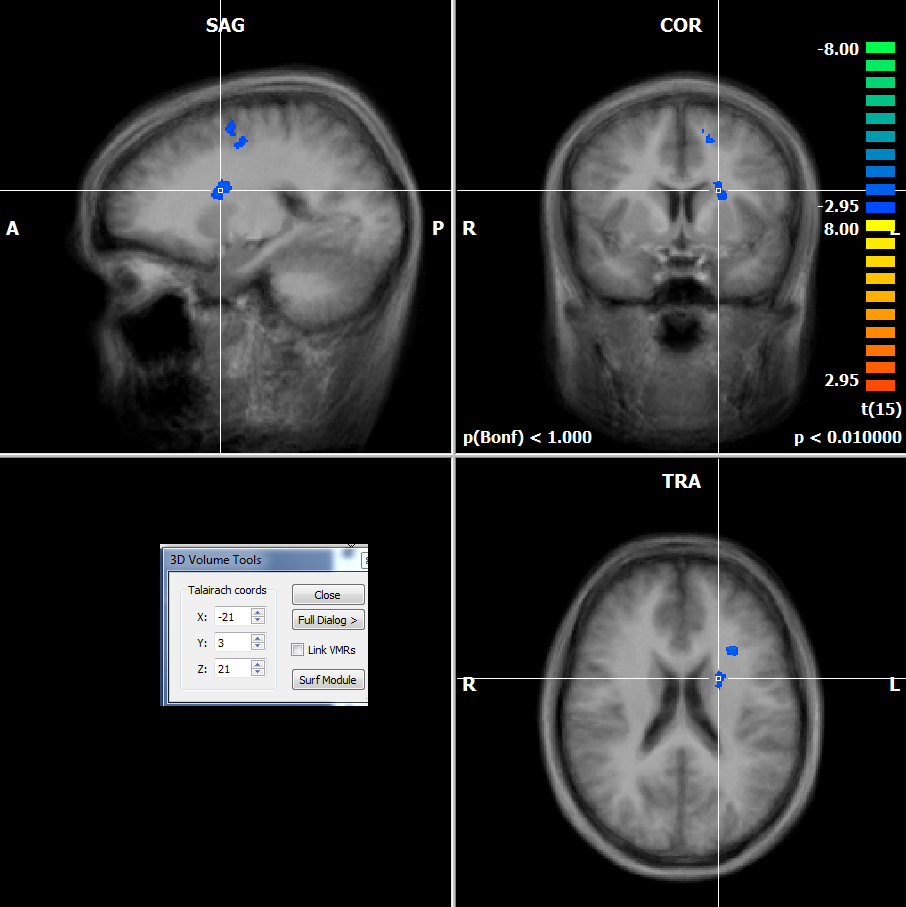
S5 Fig

Supplement: S5 Fig — (DOCX) [file pone.0198204.s005.docx]

**S6 and S7 Figs: Overview of mean errors and RTs (in ms)**

**S6 Fig**

**
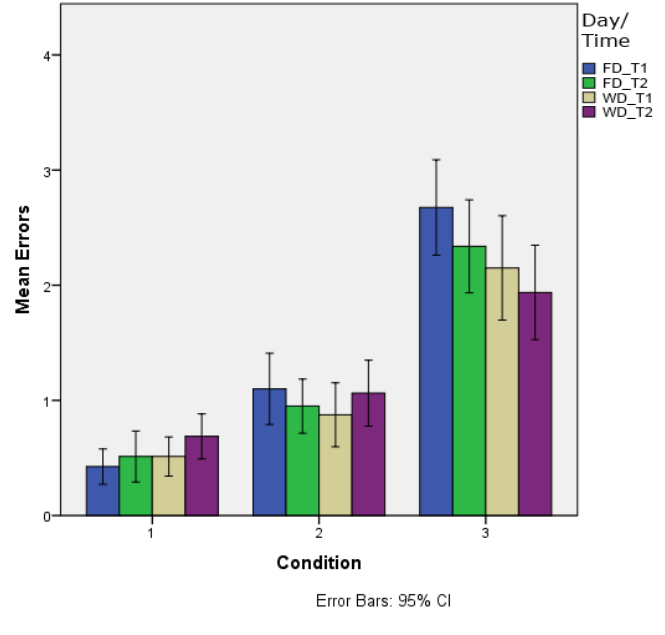
**

Supplement: S6 Fig — (DOCX) [file pone.0198204.s006.docx]

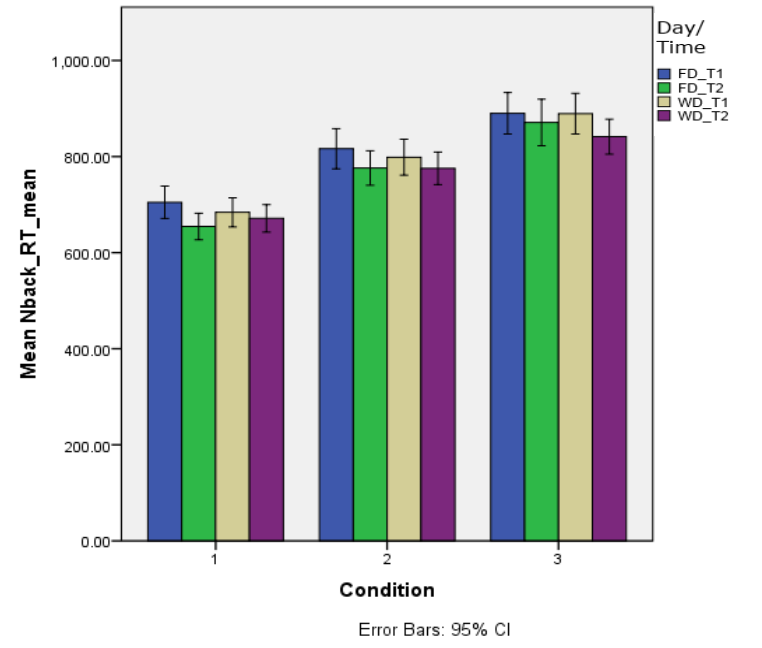
S7 Fig

Supplement: S7 Fig — (DOCX) [file pone.0198204.s007.docx]
