# Supplementary material for: Feeling the force: Changes in a left-lateralized network of brain areas under simulated workday conditions are reflected in subjective mental effort investment
Source: PLoS One. 2018 Jun 18;13(6):e0198204. doi: 10.1371/journal.pone.0198204 (PMC6005543; doi:10.1371/journal.pone.0198204)
Supplement: S2 Fig — (DOCX) [file pone.0198204.s002.docx]

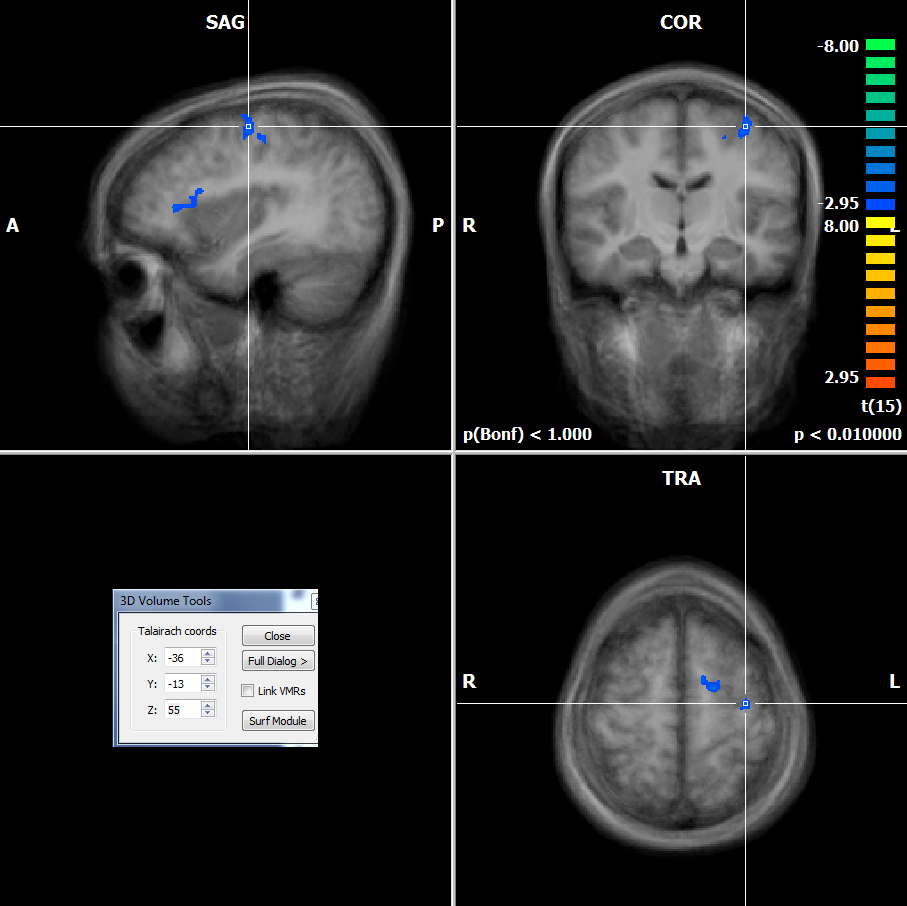
**
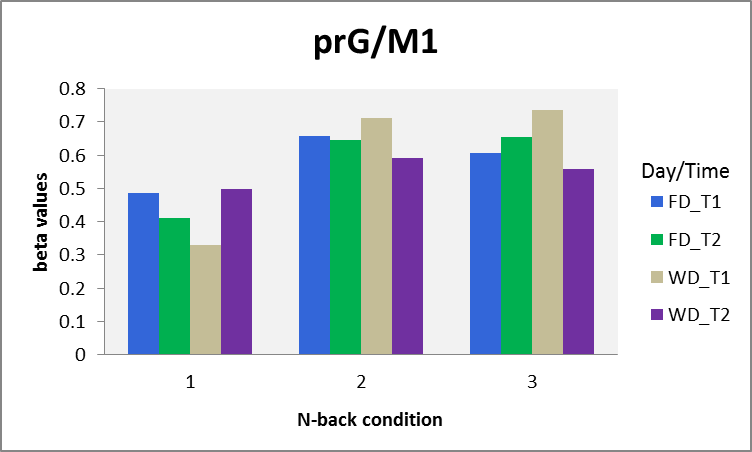
S2 Fig: Cluster in precentral gyrus / M1 and related beta values**

These frontal areas have previously been identified as part of the task-positive or cognitive control network of areas that become selectively more active in a working memory task (1). Alnaes et al. (2) reported a cluster including FEF and parts of the precentral gyrus as being sensitive to task load in a visual tracking task. Furthermore, a smaller sub-cluster including FEF and, on the left side, the inferior part of the precentral gyrus also showed a relation with pupil dilation during tracking task performance. Pupil dilation has been previously proposed as a measure of mental effort (3), and was used for that purpose by Alnaes et al. (2).

It has been proposed that, particularly under stressful conditions, a top-down regulation of attentional and sensory-motor systems takes place (4). According to the authors, this serves to enhance stimulus salience, and facilitates reacting to changing stimuli under challenging task performance conditions.

# References

1. Basten U, Stelzel C, Fiebach CJ. Intelligence is differentially related to neural effort in the task-positive and the task-negative brain network. Intelligence. 2013 Sep;41(5):517–28.

2. Alnaes D, Sneve MH, Espeseth T, Endestad T, van de Pavert SHP, Laeng B. Pupil size signals mental effort deployed during multiple object tracking and predicts brain activity in the dorsal attention network and the locus coeruleus. Journal of Vision. 2014 Apr 1;14(4):1–1.

3. Kahneman D, Tursky B, Shapiro D, Crider A. Pupillary, heart rate, and skin resistance changes during a mental task. Journal of Experimental Psychology. 1969;79(1, Pt.1):164–7.

4. Sarter M, Gehring WJ, Kozak R. More attention must be paid: The neurobiology of attentional effort. Brain Research Reviews. 2006 Aug;51(2):145–60.
